# COVID-19 Pandemic Waves and 2024–2025 Winter Season in Relation to Angiotensin-Converting Enzyme Inhibitors, Angiotensin Receptor Blockers and Amantadine

**DOI:** 10.3390/healthcare13111270

**Published:** 2025-05-27

**Authors:** Anna Puigdellívol-Sánchez, Marta Juanes-González, Ana Isabel Calderón-Valdiviezo, Helena Losa-Puig, Marta González-Salvador, Marc León-Pérez, Luís Pueyo-Antón, Maite Franco-Romero, Celia Lozano-Paz, Albert Cortés-Borra, Roger Valls-Foix

**Affiliations:** 1 Medicina de Familia, CAP Anton de Borja-Centre Universitari, c/Marconi-Cantonada Edison s/n, Consorci Sanitari de Terrassa (CST), 08191 Rubí, Spain; mjuanes@cst.cat (M.J.-G.); acalceronv@cst.cat (A.I.C.-V.); helenalosa@uic.es (H.L.-P.); mleonperez96@gmail.com (M.L.-P.); lpueyo@cst.cat (L.P.-A.); mfranco@perevirgili.cat (M.F.-R.); clozano@cst.cat (C.L.-P.); rvalls@cst.cat (R.V.-F.); 2 Human Anatomy and Embryology Unit, Faculty of Medicine, c/Casanova 143, Universitat de Barcelona, 08036 Barcelona, Spain; 3 Hospital Álvaro Cunqueiro, Estrada de Clara Campoamor 341, 36213 Vigo, Spain; 4 Management, Control and Information Analysis Unit, Hospital de Terrassa, Consorci Sanitari de Terrassa (CST), Carretera de Torrebonica s/n, 08227 Terrassa, Spain; mgonzalezs@cst.cat; 5 Independent Researcher, 08207 Sabadell, Spain; 6 Parc Sanitari Pere Virgili, c/d’Esteve Terradas, 30, Gràcia, 08023 Barcelona, Spain; 7 CAP La Garriga, c/Torrent de la Sínia 7, Institut Català de la Salut, 08530 La Garriga, Spain; alcortes.bcn.ics@gencat.cat

**Keywords:** COVID-19, ACE inhibitors, angiotensin receptor blockers, amantadine

## Abstract

**Background:** Early pandemic reports suggested improved outcomes in hypertensive COVID-19 patients treated with angiotensin-converting enzyme inhibitors (ACEI) or amantadine. This study evaluates their impact on disease progression. **Methods:** We analyzed 55,936 infected patients (March 2020–January 2025) and 2024 hospital admissions within a free-access Barcelona metropolitan health consortium (*n* = 192,651 as of March 2025). Hospitalizations, stratified by polypharmacy level (nT), were compared via Chi-square tests. ICU admissions and length of stay in hospitalized patients were assessed during the first month of key waves: initial A2a + B3a + B9 (*n* = 184, March 2020), Delta (*n* = 158, July 2021), Omicron21K (*n* = 142, January 2022), and Omicron 24F (*n* = 8, January 2025). **Results:** Non-survivors were predominantly aged >60 years (96.3%) in the first wave and >70 years (100%) in Delta/Omicron waves. Post-vaccination, mortality decreased in high-comorbidity groups, though hospitalizations/ICU admissions in younger patients surpassed first-wave levels during Delta. Vaccinated ACEI/ARB-treated patients showed reduced hospitalizations across all polypharmacy groups: OR (noACEI/ACEI) = 1.21 (≥2 nT) to 4.26 (1 nT, *p* = 0.014); OR (noARB/ARB) = 1.24 (≥8 nT) to 1.74 (2–7 nT, *p* = 0.01). No hospitalizations occurred in amantadine-treated patients aged <70. Conclusions: These findings suggest a potential protective effect of ACEI, ARBs, and amantadine against severe COVID-19 and support the safety and continuity of these treatments. Multicentric studies incorporating post-COVID syndrome data are needed to validate these observations if hospitalizations persist.

## 1. Introduction

Potential interventions to treat COVID-19 were proposed early in the pandemic. Among these, angiotensin-converting enzyme inhibitors (ACEIs) and angiotensin receptor blockers (ARBs) were reported to improve clinical outcomes in COVID-19 patients with hypertension [1]. However, initial support for this claim relied primarily on limited experimental data, such as studies demonstrating the protective effect of ACEIs on neurogenic pulmonary edema [2]. Concerns were later raised about their use, as these drugs might upregulate ACE2—the receptor for SARS-CoV-2—potentially increasing viral susceptibility [3]. Nonetheless, subsequent evidence showed that these concerns were unfounded [4,5].

Other potential targets for drug repurposing were explored early in the pandemic following the cloning of SARS-CoV-2 proteins, with some showing high-confidence interactions with human proteins [6]. Among the 29 FDA-approved drugs identified as modulators of host factors interacting with the virus was Entacapone, a catechol O-methyltransferase inhibitor used for Parkinson’s disease. Preliminary observations of mild infections in vulnerable patients [7], along with later randomized controlled trials on post-COVID syndrome [8], suggested that amantadine might also alleviate severe COVID-19 symptoms. Other candidate drugs for repurposing included the antihistamine loratadine [6]. Antihistamines were associated with improved outcomes in observational studies [9]. Notably, the pragmatic administration of antihistamines during the first wave in nursing homes avoided hospitalizations and deaths [10]. When applied in primary care settings (e.g., Yepes, Spain), it decreased both hospital admissions and the incidence of long-term COVID syndrome [11].

Nirmatrelvir is currently the only approved drug for early COVID-19 treatment in elderly patients, having demonstrated reduced hospitalizations, ICU admissions, and mortality in high-risk populations [12]. However, evidence shows it neither prevents infection in COVID-19 contacts [13] nor reduces progression to long-term COVID [14]. Additionally, its high cost and potential for drug interactions limit widespread use.

Previous research by our group demonstrated that polypharmacy—rather than any specific pharmacological class—was associated with worse COVID-19 outcomes [15]. This finding was supported by the observation that early admitted patients during the first wave peak used up to 91 different medications [9]. Notably, frailty [16], which is closely correlated with polypharmacy [17], emerged as a significant predictor of severe COVID-19 symptoms. Consequently, any assessment of potential drug-related protective effects must account for polypharmacy as a critical confounding variable.

The profile of COVID-19 patients who needed hospital admission during the winter of 2024–2025 is described here and compared with data on polypharmacy and vaccination from the previous first, Delta, and Omicron pandemic waves, considering that the effect of any tested drug must also consider the different COVID evolution under the various pandemic and post-pandemic variants. The association of several chronic treatments—ACEI therapy, ARBs, and amantadine—with better COVID outcomes will be explored here, with the aim of preparing future prospective interventional studies after early detection of SARS-CoV-2 infection in primary care or to justify potential compassionate use after hospital admission.

## 2. Materials and Methods

The study was approved by the Ethics Committee of the Consorci Sanitari de Terrassa (CST) on 8 April 2020 (Ref: 02-20-161-021). The observational clinical trial was registered on 29 April 2020 (NCT04367883). The planning, conducting, and reporting of the study adhered to the principles of the Declaration of Helsinki and EU Regulation 2016/679. The initial study, focused on hospital admissions, was expanded to include antihistamines on 18 October 2021, and later amantadine, with these factors linked to the reference population on 22 June 2022 (https://clinicaltrials.gov/study/NCT05504057; accessed on 1 December 2024). Following the inclusion of the primary care center ‘CAP Can Roca’ into the CST, the retrospective study of the current population (*n* = 192,651), including post-COVID syndrome, was approved on 24 February 2025.

### 2.1. Socioeconomic Environment

The details of the socioeconomic characteristics of the CST have been published previously. In summary, the CST is a free public integral health organization in the North Metropolitan Barcelona Health Region. This health organization includes eight primary healthcare centers, one long-term care center, and the Hospital of Terrassa, which serves as a referral center for hospitalization.

Centers are located in rural (Castellbisbal) and residential villages (Matadepera) or metropolitan cities (Terrassa and Rubí), and the population is within less than 30 min by car from the Hospital de Terrassa. Despite variable socioeconomic factors among centers (pre-pandemic incomes < EUR 18,000 ranging from 58% to 67% among centers, with incomplete primary education affecting 16.9–26.6% of the population), the different centers showed a pre-pandemic life expectancy above 81 years, comparable COVID19 vaccination rates above 90% in patients over 60 years old with 2 or more chronic prescriptions, and pandemic infection rates between 22% and 27% [9].

The rates of prescription of the different studied drugs were quantified here.

### 2.2. Quantification of Cases and Hospitalizations

The Data Analysis Control Department collected anonymized data for COVID-19 cases and hospitalizations, together with data on gender, age, number of chronic treatments (nT)—including or not ACEIs, ARBs, and amantadine—and their COVID-19 vaccination status before the first infection in the CST population. Deceased COVID-19 hospitalized patients were reviewed for comorbidities (Charlson index) following Regulation (EU) 2016/679.9. Pandemic cases were studied previously by our group in the context of the population existing in March 2020 [9,15]. Now, the CST population in March 2025 was analyzed retrospectively.

The presence of COVID-19 was determined either by polymerase chain reaction (PCR), an antigen test, or clinical criteria (compatible interstitial pneumonia) in hospital-admitted patients.

In primary care, cases were confirmed by PCR after 1 June 2020, and either by PCR or antigen test between November 2020 and 23 March 2022, in suspected cases and close contacts. These protocols ended after 23 March 2022. Between 1 March and 30 May 2020, cases were recorded based on clinical suspicion, as tests were not available.

The date of COVID-19 vaccination was used to classify patients as vaccinated or unvaccinated prior to the first infection. Age was adjusted to the date of the first COVID-19 infection.

### 2.3. Vaccination

COVID-19 vaccinations with Pfizer^®^ [18] and Moderna^®^ [19], began in 2021, starting with older people, while AstraZeneca^®^ [20] and Janssen^®^ [21] vaccines were administered to younger age groups.

Vaccination was considered complete in July 2021 if one dose of Janssen^®^ or two doses of any other COVID-19 vaccine had been received. The recombinant Novavax vaccine [22] was only administered in cases of severe reactions to mRNA vaccines. For Omicron waves, vaccination was considered complete if patients had been vaccinated during all previous autumn campaigns.

Patients who had not received the vaccine prior to their first infection were grouped in tables with unvaccinated patients to separate the likelihood of hospital admission between vaccinated and unvaccinated individuals.

### 2.4. COVID-19 Variants

The web Covariants.org [23] was consulted to determine the predominant variant in our region during each period with hospitalization peaks. When a hospitalization peak coincided with a predominant variant, that month was selected for comparison with other waves. Thus, the first wave coincided with an evolution of circulating variants that became a specific profile for Spain [24], producing a peak in hospitalizations and deaths in March 2020. This wave has been compared here with the Delta variant, which caused hospitalization peaks in July 2021, and with the first Omicron variant that led to hospitalization peaks in January 2022 (21 K).

Variant 24E was predominant during autumn of 2024 in Spain. The last post-pandemic wave corresponded with Variant 24F, which became the final predominant variant in December 2023 after coexisting with Variant 24E during October and November.

According to the Catalan website for weekly epidemiological data, the currently circulating variants as of March 2025 are XEC and KP.3, detected in 33% of randomly selected sentinel samples [25].

### 2.5. Intensive Care Unit Needs and Length of Hospital Stay

Since Consorci Sanitari de Terrassa is an integrated healthcare organization, a single patient might be admitted to the emergency department, hospitalized, later transferred to the ICU, and—depending on frailty—either continue in-house hospitalization or be referred to an external ICU before readmission. To calculate total hospital stay length, sequential hospitalizations across different facilities (within the same organization or related to the same infection) were combined and measured from emergency room admission until final home discharge (with no further intravenous treatments).

### 2.6. Statistical Analysis

Numbers of COVID-19-infected and hospitalized patients were analyzed, excluding reinfections and multiple hospital admissions of the same patient. Statistical analyses used OpenEpi resources (open-source epidemiologic statistics for public health) [26].

Patients were stratified by age (≤60 or >60 years) and number of treatments (nT), based on observed mortality associations with polypharmacy rather than specific pharmacological profiles [9,15]. Age averages per nT were calculated in wave comparison tables. Post-massive vaccination reductions in hospitalizations and mortality [15] prompted additional stratification by pre-infection vaccination status.

Subgroups (based on age/nT/vaccination status) were compared using OpenEpi’s Chi-square tests [27] for infection/hospitalization rates in 2 × 2 drug exposure tables. For subgroups with <5 cases, Fisher’s exact test [28] was applied. Chi-square tests and Fisher’s exact tests are the methods recommended by OpenEpi resources for unmatched case–control studies, where a single exposure variable (expressed as multiple ordered categories) is compared across multiple control groups [26]. This approach followed the same methodology used in prior studies of antihistamines’ protective effects [15].

## 3. Results

### 3.1. Socioeconomic Environment and Prescription

Detailed population and prescription data for centers within the CST are presented in Table 1. The distribution shows comparable proportions of patients >60 years old (15–24%) and similar prescription rates for ACEI (32–36%) and ARBs (11–13%). These patterns were consistent across metropolitan cities, representing 89% of the CST population

### 3.2. Quantification of Cases and Hospitalizations During the Pandemic Period and Last Winter Season 2024–2025

**Figure 1:**
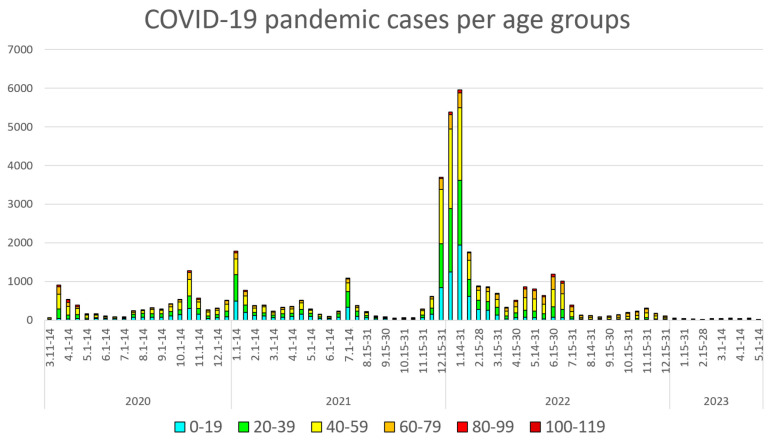
COVID-19 case distribution by age group (20-year intervals) and time period (biweekly intervals) during the pandemic.

**Figure 2:**
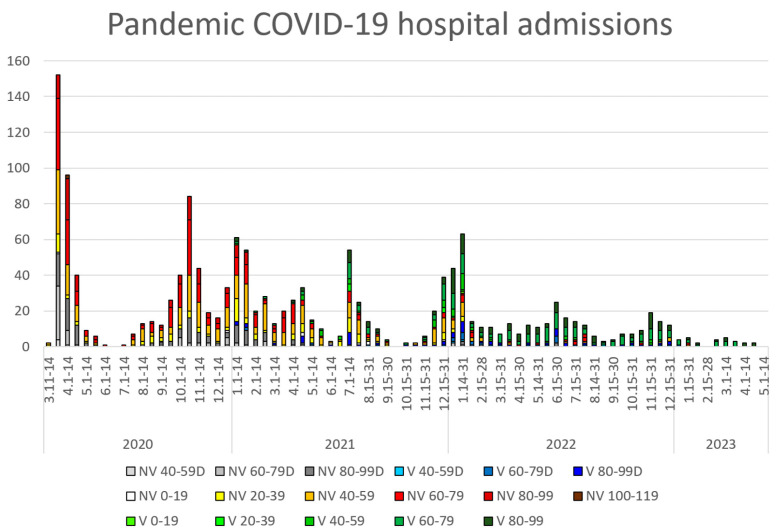
COVID-19 hospital admissions and deaths (D) by vaccination status (V = vaccinated, NV = non-vaccinated) and age group (20-year intervals), shown in biweekly periods during the pandemic.

Figure 1 illustrates registered COVID-19 cases during the pandemic period, stratified by age groups, while Figure 2 presents corresponding hospital admissions.

**Figure 3:**
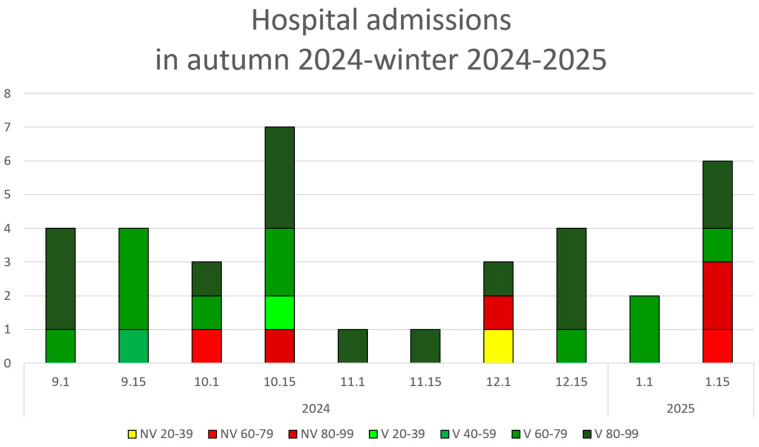
COVID-19 hospital admissions stratified by vaccination status (V = vaccinated; NV = non-vaccinated), age groups (20-year intervals), and biweekly periods from September 2024 until the last recorded admission of the winter on 27 January 2025.

Post-pandemic hospitalization data (autumn 2024 through winter 2025) are shown in Figure 3.

Following a COVID-19 hospitalization on 27 January 2025, no further hospitalizations were recorded throughout February 2025. The next admission occurred on 27 March 2025, involving a 30-year-old male patient with only one chronic treatment and no documented vaccination history.

### 3.3. Comorbidity in the First Fatal Cases

The search for a specific comorbidity profile among the first 63 deceased cases during the initial pandemic month revealed hypertension (67%), dyslipidemia (48%), diabetes mellitus (29%), obesity (31%), and current smoking (54.4%) as predominant factors. Additionally, chronic obstructive pulmonary disease (COPD) was present in 24.2% of cases, while asthma affected only 1.6%. The mean glomerular filtration rate prior to final hospital admission was 62.8 ± 22.6 mL/min.

The average Charlson Comorbidity Index was 2.7 ± 2.9. Analysis of its components showed 29% had diabetes with end-organ damage, 24.6% congestive heart failure, 15% peripheral vascular disease, 14.8% prior myocardial infarction, 24.2% cerebrovascular disease, 16.1% dementia, and 12.9% hemiplegia. Additionally, 39.3% had metastatic cancer, 3.2% had leukemia, 4.8% had connective tissue disorders or peptic ulcer disease, and 6.6% had moderate-to-severe liver disease.

Deceased patients had an average of 8.05 ± 4.34 chronic treatments, encompassing 70 distinct drug classes. The most frequently prescribed medications (prevalence > 20%) included: proton pump inhibitors (54.7%), statins (40.6%), diuretics (37.5%), benzodiazepines (35.9%), analgesics (34.4%), β-blockers (29.7%), selective serotonin reuptake inhibitors (28.1%), and β_2_-agonists (23.4%).

A total of 25.4% of those fatal cases had ACEI as chronic treatment, and 17.5% received ARB, compared to the 34% of ACEI (*p* = 0.06) and 11.4% of ARB (*p* = 0.07) in the population over 60 years old in the CST in April 2020.

### 3.4. Polypharmacy, Hospitalization, and ICU in the Main Pandemic Waves and in Post-Pandemic Winter 2024–2025

During the first wave, polypharmacy and age showed direct associations with survival outcomes, while vaccination status significantly modified mortality among fragile patients (those with >8 chronic prescriptions). Notably, 57% (28/49) of hospital admitted patients in this polypharmacy group died within one month during the initial wave, compared to only 39.5% (13/33) mortality during Omicron (despite similar admission criteria). By January 2025, just two patients with ≥8 chronic treatments were hospitalized, with one fatal outcome (Table 2).

A progressive increase in admitted patients’ age was observed across pandemic waves, accompanied by reduced hospital stays and ICU demand. During the first wave, >10% of all admitted patients required ICU care, peaking at 25% among younger chronic treatment recipients during the Delta wave (August 2021)–when vaccination coverage remained low in under-50 populations (initiated just one month prior). In contrast, highly vaccinated (≥90% full vaccination) fragile patients (≥8 nT) showed significantly better outcomes, with 85.7% survival and no ICU needs.

The two fatal COVID-19 cases in January 2025 involved vaccinated males (aged 88 and 74 years)—one with 12 chronic medications and six vaccine doses (last administered October 2024), the other with six chronic treatments, and a final dose in October 2023. These represented the only COVID-19-related deaths at our institution since September 2023.

No patients treated with amantadine required hospitalization during the first pandemic wave. Notably, no COVID-19-related deaths occurred among amantadine recipients throughout the entire study period (March 2020–January 2025).

### 3.5. Relationship Between Polypharmacy and Drug Repurposing: ACEI-ARB and Amantadine

COVID-19 infection and hospitalization rates (March 2025) in the CST population are presented for the three studied drug groups (ACE inhibitors, ARBs, and amantadine).

As most ACEI/ARB recipients were >60 years old, demographic groups with the highest hospitalization risk-odds ratios (ORs) were calculated within this specific age strata to control for this confounding factor.

#### 3.5.1. ACEI

Among the CST population aged over 60 years, untreated with ACEI, the vaccinated group showed significantly higher hospitalization rates compared to those treated with ACEI alone (OR 4.26; *p* = 0.014). Other comparison groups exhibited non-significant but consistent increases in rates (OR range: 1.12–1.55), except for the non-vaccinated group receiving 2–7 chronic treatments (OR = 0.99) (Table 3).

#### 3.5.2. ARB

In the CST population > 60 years old, ARB-untreated vaccinated patients showed higher hospitalization rates versus ARB-treated peers, with OR = 1.24 in severe polypharmacy (nT ≥ 8) and OR = 1.74 (*p* = 0.01) for those receiving 2–7 chronic treatments. No vaccinated patients on ARB monotherapy required hospitalization. Non-vaccinated patients showed neutral associations (OR range 0.82–1.05) without trends related to the number of treatments (Table 4).

#### 3.5.3. Amantadine

Amantadine treatment was administered to 52 patients in our institution, with 50 patients (96.2%) aged 40–85 years. Two outliers were excluded from the analysis: one 23-year-old and one 92-year-old. For reporting, patients were stratified into 15-year age groups starting at >40 years, with additional subgroups for patients < 40 years and those >85 years.

No hospital admissions occurred in amantadine-treated patients aged <70 years. Only two vaccinated patients (aged 78 and 84) required hospitalization. After Fisher’s exact test correction for small sample sizes, no significant differences were observed between groups in case severity or hospitalization rates (Table 5 and Appendix A).

## 4. Discussion

The concerted health consortiums cover the pharmacy costs of prescriptions issued by their employed physicians. The adherence of chronic prescriptions to evidence-based general prescribing guidelines, as well as the degree of hypertension control across participating institutions, is periodically reviewed by healthcare authorities. Consequently, the IT infrastructure is designed to automatically generate prescription data for a given population without requiring manual checks of individual medical records or other registries. Overall, the data obtained directly from the Data Analysis Control Department—pertaining to the entire CST population—is regarded as highly reliable.

### 4.1. Statistical Approach Relating Polypharmacy and Age

The analysis of COVID-19 naïve patients hospitalized during the first month revealed a linear relationship between mortality and the number of chronic treatments. Polypharmacy was also associated with hospitalization in all other explored waves [15]. However, the specific study of polypharmacy in the first deceased patients detected up to 70 different types of medications without any specific pathological pattern. Furthermore, patients without any known chronic conditions were also hospitalized during all the pandemic waves studied.

Altogether, the linear and consistent association between the number of chronic treatments, age, and hospital admissions across all waves led us to group patients using those parameters. The age stratification (≥60 vs. <60 years) was chosen because >90% of first-wave mortality occurred in patients over 60. The treatments studied were then analyzed in relation to polypharmacy in patients above and below 60 years old.

Chi-square tests [27], complemented by Fisher’s exact test when the number of individuals in a category is small, assess independence (as opposed to association) in 2 × 2 contingency tables. Here, the dichotomized attributes correspond to treatments (treated or not treated with the studied drugs) and the response to those treatments (cases or hospitalization) [28]. These tests were repeated for each subgroup stratified by polypharmacy and age, as performed previously for the assessment of antihistamine protective effects [9].

### 4.2. ACEI and ARB

This manuscript is the first to report a reduced hospitalization rate among vaccinated patients with chronic prescriptions for ACE inhibitors (ACEIs) and angiotensin receptor blockers (ARBs)—commonly prescribed in primary care, either alone or alongside other chronic medications. Notably, significant results were observed when these drugs were taken alone (monotherapy [1 nT] for ACEIs) or with a limited number of concurrent chronic prescriptions (no hospitalizations occurred with 1 nT, and rates were significantly reduced for 2–7 nT in the ARB group). Additionally, low *p*-values were detected in other polypharmacy groups within the ACEI comparison, suggesting that statistical significance might be achieved with a larger sample size.

Randomized controlled trials conducted during the early stages of the pandemic did not support discontinuing ACEI or ARB use in hospitalized patients with mild to moderate COVID-19 [29]. Subsequent studies reported an increased risk of heart failure when these treatments were interrupted [30]. While newer research demonstrated improved outcomes (specifically, more days alive and out of the hospital at 30 days) [31], other studies found no benefits and even potential worsening when these therapies were initiated during hospitalization [32]. Notably, all these trials were conducted in hospital settings, by which point SARS-CoV-2 infection severity had already progressed.

Multicenter studies are needed to assess whether reduced hospitalization rates persist across all polypharmacy groups receiving chronic ACEI or ARB therapy in primary care.

### 4.3. Amantadine Prescription

While amantadine has demonstrated inhibitory effects against SARS-CoV-2 in vitro, initial enthusiasm has been tempered by subsequent errata [33]. Preliminary randomized controlled trials evaluating amantadine as an adjunct therapy in hospitalized patients showed no improvement in recovery rates [34,35].

In outpatient settings, prospective clinical trials have reported that amantadine may alleviate post-COVID syndrome symptoms, including fatigue [8], as well as sleep disturbances and depression [36].

The limited number of chronic patients receiving this treatment precluded conclusive results. However, the absence of hospital admissions among patients under 70 years old appears promising. Multicenter studies remain necessary to verify whether a significant reduction in hospitalizations proves consistent, while prospective trials should evaluate its potential as early intervention in selected patient populations.

### 4.4. Limitations of the Study

The initial 2020 study population did not include patients from ‘CAP Can Roca’, which joined the CST consortium on 3 October 2022. Consequently, COVID-19 infections, vaccinations, and hospital admissions prior to this date may be underestimated. However, CAP Can Roca serves a population of 20,730 individuals, representing just 11% of the total institutional population, suggesting minimal impact on overall data interpretation. Additionally, immigration patterns have contributed to CST’s growing patient base. This accounts for the disparity between the 2020 ambispective follow-up population (approximately 160,000) [9,15] and the current retrospectively analyzed population (approximately 190,000). On the other hand, up to 2585 patients from the original March 2020 cohort had died by the end of the pandemic without COVID-19-related hospitalizations [9]. However, approximately 40% of those over 60 years old were likely treated with ACEIs or ARBs. This may introduce bias into the retrospectively studied population and reduce the sensitivity of detecting the protective effect against hospitalizations in the non-vaccinated population (as many of these deaths occurred prior to vaccine availability).

In Spain, COVID-19 vaccination was initially administered through primary care centers beginning in early 2021, prioritizing older populations. After June 2021, vaccination became available to all age groups at various centers, including locations outside the CST network. This decentralized approach may have resulted in the underreporting of vaccinations among younger populations in CST records. Nevertheless, vaccination coverage exceeds 90% across all CST centers for high-risk patients (i.e., those aged > 60 years receiving ≥ 2 chronic treatments)—the same population in which the positive effect of ACEIs and ARBs was detected [9].

Within the same district, additional private and publicly subsidized private hospitals exist—specifically Hospital General de Catalunya and Hospital Mútua de Terrassa—where assigned population patients may also be admitted. Therefore, the recorded hospitalization rates likely represent an underestimate of true figures.

Private consultations could theoretically lead to underreporting of ACEI, ARB, or amantadine prescriptions. However, treatment cost subsidies typically prompt patients to consult their assigned public primary care physician, who generally incorporates these medications into the public electronic chronic prescription system when approving the private treatment. We assumed equivalent therapeutic adherence across all study groups.

### 4.5. Present and Future

Current surveillance data from the Catalan epidemiological network indicate that variants XEC and KP.3 comprise 33% of circulating strains, as identified in randomly selected sentinel samples [25]; however, hospitalization rates remain at baseline levels in the region. WHO reports confirm declining trends, with documented reductions in both COVID-19 cases [37] and mortality [38] over the past month. While periodic post-pandemic admission surges have occurred at magnitudes comparable to peak pandemic levels, the 2024–2025 winter season saw unusually low COVID-19 hospitalizations despite concurrent influenza virus resurgence [25].

Should these low hospitalization rates persist, our findings provide valuable documentation of the epidemiological evolution of the pandemic.

## 5. Conclusions

A significant reduction in hospital admissions has been observed in several groups of COVID-19-vaccinated patients treated with ACEI or ARB, alongside an absence of hospitalizations in amantadine-treated patients under 70 years old. Further multicentric retrospective studies are needed to confirm the consistency of the reduction in hospital admissions across all studied drugs, while prospective trials might confirm their protective role.

## Figures and Tables

**Table 1:** Demographic data from primary care centers within the CST reveal consistent treatment patterns among patients >60 years old, with comparable prescription percentages for ACEIs, ARBs, and Amantadine.

	Assigned	% CST	>60 Years Old			
Metropolitan Cities	Population	Population	%	% ACEI	% ARB	% Amantadine
Terrassa-CAP SANT LLATZER	45,061	23.4%	23.6%	33.3%	11.3%	0.11%
Terrassa-CAP TERRASSA EST	28,950	15.0%	15.1%	36.0%	11.6%	0.05%
Terrassa-CAP TERRASSA NORD	27,573	14.3%	22.7%	34.1%	11.3%	0.06%
Terrassa-CAP CAN ROCA	21,482	11.2%	22.5%	34.8%	12.1%	0.12%
Rubí-CAP ANTON DE BORJA	31,696	16.5%	22.4%	34.5%	13.4%	0.07%
Rubí-CAP SANT GENIS	16,405	8.5%	21.0%	32.4%	11.2%	0.00%
**Residential or rural villages**						
Castellbisbal-CAP DR JOAN PLANAS	11,459	5.9%	19.4%	35.3%	7.9%	0.04%
CAP MATADEPERA	10,025	5.2%	24.0%	27.0%	10.4%	0.12%

**Table 2:** Polypharmacy and age in the first month of the main COVID-19 waves, with data on ACEI and ARB treatments and hospital stay characteristics.

Number of Chronic Treatments-nT- (*n* Patients)	Age	Gender, % Females	% ACEI	% ARB	SARS-CoV-2 Vaccination (Complete)	Length of Hospital Stay	ICU	Non-Survivors
**March 2020 (A2a + B3a + B9)**								
0 nT (*n* = 26)	45.3 ± 16.0	30.7%	0%	0%	0	13.9 ± 15.1	13.9%	7.9%
1 nT (*n* = 14)	65.2 ± 12.9	28.5%	14.2%	7.1%	0	12.2 ± 9.8	12.2%	21.4%
2–4 nT (*n* = 51)	63.6 ± 15.2	43.4%	30.2%	9.4%	0	18.3 ± 20.0	18.3%	16.9%
5–7 nT (*n* = 42)	70.6 ± 10.4	35.7%	31.0%	21.4%	0	17.6 ± 15.7	21.9%	30.9%
>8 nT (*n* = 49)	75.2 ± 11.6	49.0%	20.4%	24.5%	0	16.0± 16.0	10.2%	57.1%
**July 2021 (Delta)**								
0 nT (*n* = 59)	37.7 ± 10.7	31.0%	3.4%	0.0%	31.7% (5.1%)	8.1 ± 6.1	25.4%	1.7%
1 nT (*n* = 20)	44.3 ± 15.9	45.0%	10.0%	5.0%	25.0% (10.0%)	10.2 ± 16.3	10.0%	0.0%
2–4 nT (*n* = 33)	54.0 ± 17.8	48.5%	12.1%	12.1%	42.4% (33.3%)	9.5 ± 5.0	9.1%	3.0%
5–7 nT (*n* = 18)	70.0 ± 16.2	55.6%	33.3%	29.4%	44.4% (27.8%)	12.8. ± 14.0	22.2%	17.0%
>8 nT (*n* = 28)	77.9 ± 19.5	60.7%	25.0%	14.3%	96.4% (92.8%)	13.8 ± 10.3	0.0%	14.3%
**January 2022 (Omicron21K)**								
0 nT (*n* = 36)	54.7 ± 25.2	44.4%	13.9%	2.8%	36.1% (36.1)	8.3 ± 6.6	8.3%	2.8%
1 nT (*n* = 10)	55.7 ± 16.3	30.0%	40.0%	0.0%	50.0% (50%)	9.4 ± 5.4	20.0%	0.0%
2–4 nT (*n* = 41)	66.5 ± 17.1	51.2%	36.6%	7.3%	48.8% (48.8%)	10.3 ± 9.2	2.4%	2.4%
5–7 nT (*n* = 22)	72.4 ± 14.7	27.8%	68.2%	9.1%	63.6% (63.6%)	8.3 ± 4.5	0%	18.2%
>8 nT (*n* = 33)	79.9 ± 11.1	48.5%	45.5%	9.1%	78.8% (45.5%)	8.8 ± 5.9	3.0%	39.4%
**January 2025 (Omicron24F)**								
2–4 nT (*n* = 3)	77.6 ± 4.5	66.6%	33.3%	66.6%	100% (66.6%)	5.6 ± 1.1	0%	0%
5–7 nT (*n* = 3)	82.6 ± 14.1	33.3%	66.6%	0%	66% (33%)	8.6 ± 1.1	0%	33%
≥8 nT (*n* = 2)	87.5 ± 0.7	50.0%	100%	0%	50% (50%)	5.5 ± 2.1	0%	50%

**Table 3:** COVID-19-related hospitalizations in CST patients >60 years old (observation period: March 2020–March 2025), categorized by chronic treatment burden (nT) and ACEI use status. Results present hospitalization odds ratios (ORs) with statistical significance indicated (* *p* < 0.05). The NoVAC group combines unvaccinated individuals and those infected prior to initial vaccination.

	NoVAC Pre-Infection		*p Hospitalization*	VAC Pre-Infection		*p Hospitalization*
	ACEI	No ACEI	OR NoACEI/ACEI	ACEI	No ACEI	OR NoACEI/ACEI
Ages 0–59	3231	98,035		3499	46,603	
Ages ≥ 60	2036	6530		11,891	20,826	
**0 nT**		**2922**			**3636**	
Cases		397			466	
Hospitalization		46			11	
**1 nT**	**215**	**855**		**699**	**2517**	
Cases	66	227	*p = 0.34*	84	454	p = 0.014 *
Hospitalization	6	32	1.55	1	16	4.26
**2–7 nT**	**1417**	**2366**		**7946**	**11674**	
Cases	632	1064	*p = 0.96*	1537	2481	*p = 0.07*
Hospitalization	132	220	0.99	89	159	1.21
**≥8 nT**	**404**	**387**		**3247**	**2999**	
Cases	300	306	*p = 0.40*	821	806	*p = 0.07*
Hospitalization	82	88	1.12	97	109	1.21

**Table 4:** Hospital admissions among CST patients aged > 60 years with COVID-19 infection (10 March 2020–28 March 2025), stratified by the total number of chronic treatments (nT), with or without ARB. Odds ratios (ORs) for hospitalization are shown with *p*-values (* *p* < 0.05). NoVAC corresponds to non-vaccinated patients, grouped with patients infected before receiving the first vaccine.

	NoVAC Pre-Infection		*p Hospitalization*	VAC Pre-Infection		*p Hospitalization*
	ARB	No ARB	OR NoARB/ARB	ARB	No ARB	OR NoARB/ARB
Ages 0–59	653	100,613		807	49,295	
Ages ≥ 60	704	7862		4066	28,651	
**0 nT**		**2931**			**3653**	
Cases		401			465	
Hospitalization		47			11	
**1 nT**	**60**	**1001**		**175**	**3023**	
Cases	17	272	*p = 0.89*	38	501	
Hospitalization	2	35	1.05		17	
**2–7 nT**	**499**	**3284**		**2592**	**17,028**	
Cases	249	1447	*p = 0.15*	503	3515	p = 0.01 *
Hospitalization	55	297	0.82	20	228	1.74
**≥8 nT**	**145**	**646**		**1299**	**4947**	
Cases	116	490	*p = 0.97*	344	1283	*p = 0.11*
Hospitalization	31	139	1.01	36	170	1.24

**Table 5:** Analysis of COVID-19 hospitalizations in amantadine-treated CST patients > 60 years (study period: March 2020–March 2025), stratified by chronic treatment burden (nT) and amantadine exposure status. The NoVAC cohort includes both vaccine-naïve patients and those infected prior to primary vaccination series initiation.

	NoVAC Preinf		VAC Preinf	
Ages	Amantadine	No Amantadine	Amantadine	No Amantadine
**0–39**		**63,519**	**1**	**27,635**
Cases		18,624	0	7661
Hospitalization		221	0	28
**40–54**	**4**	**30,882**	**12**	**16,186**
Cases	3	10,515	5	5703
Hospitalization	0	446	0	46
**55–69**	**4**	**12,802**	**9**	**19,998**
Cases	3	3791	3	4688
Hospitalization	0	446	0	147
**70–84**	**1**	**2300**	**19**	**14,989**
Cases		1049	6	2943
Hospitalization		270	2	226
**≥85**		**320**	**2**	**3968**
Cases		162	1	779
Hospitalization		50	0	142

## Data Availability

The original contributions presented in this study are included in the article. Further inquiries can be directed to the corresponding author.

## Abbreviations

The following abbreviations are used in this manuscript:

ACEI	Angiotensin-converting enzyme inhibitors
CST	Consorci Sanitari de Terrassa (Terrassa Health Consortium)
ARB	Angiotensin receptor blocker
nT	Number of chronic treatments
OR	Odds ratio

## Appendix A

Analysis of COVID-19 hospitalizations in amantadine-treated CST patients in all age groups (study period: March 2020–March 2025), stratified by chronic treatment burden (nT) and amantadine exposure status. The NoVAC cohort includes both vaccine-naïve patients and those infected prior to primary vaccination series initiation.

	**Amantadine**			**No Amantadine**		
	**VAC** **Post-Infection**	**VAC** **Pre-Infection**	**NoVAC**	**VAC** **Post-Infection**	**VAC** **Pre-Infection**	**NoVAC**
**0–39**	**0**	**1**	**0**	**4704**	**27,635**	**58,815**
**40–55**	**1**	**12**	**3**	**2820**	**17,437**	**29,609**
0	0	2	1	1078	8584	20,668
1	0	1	0	563	3076	4266
2–7	1	6	0	1085	5394	4563
Cases	1	3	0	1085	2044	1645
Hospital admission	0	0	0	118	28	58
≥8	0	3	2	94	383	112
Cases	0	1	1	94	157	49
Hospital admission	0	0	0	13	4	2
**56–70**	**2**	**10**	**2**	**1981**	**19,928**	**9604**
0	0	0	0	281	3924	4770
1	0	0	0	253	2998	1538
2–7	1	4	1	1192	11,218	3087
Cases	1	1	0	1192	2725	599
Hospital admission	0	0	0	244	100	43
≥8	1	6	1	255	1788	209
Cases	1	3	1	255	513	61
Hospital admission	0	0	0	65	36	8
**71–85**	**0**	**19**	**1**	**832**	**14,309**	**1185**
0	0	0	0	35	947	407
1	0	0	0	45	952	120
2–7	0	7	1	458	8973	574
Cases	0	3	0	458	1679	78
Hospital admission	0	1	0	105	103	11
≥8	0	12	0	294	3437	84
Cases	0	3	0	294	911	21
Hospital admission	0	1	0	90	109	9
**>85**	**0**	**1**	**0**	**110**	**3467**	**163**
Total general	3	43	6	10,447	82,776	99,376
